# Chalcone-imidazolone conjugates induce apoptosis through DNA damage pathway by affecting telomeres

**DOI:** 10.1186/1475-2867-11-11

**Published:** 2011-04-25

**Authors:** M Janaki Ramaiah, SNCVL Pushpavalli, G Rama Krishna, Pranjal Sarma, Debasmita Mukhopadhyay, Ahmed Kamal, Utpal Bhadra, Manika Pal Bhadra

**Affiliations:** 1 Division of Chemical Biology, Indian Institute of Chemical Technology, Tarnaka, Hyderabad-500607, India; 2 Division of Organic Chemistry, Indian Institute of Chemical Technology, Tarnaka, Hyderabad-500607, India; 3 Division of Functional Genomics and Gene silencing, Centre for Cellular and Molecular Biology, Tarnaka, Hyderabad-500007, India

## Abstract

**Background:**

Breast cancer is one of the most prevalent cancers in the world and more than one million women are diagnosed leading to 410,000 deaths every year. In our previous studies new chalcone-imidazolone conjugates were prepared and evaluated for their anticancer activity in a panel of 53 human tumor cell lines and the lead compounds identified were 6 and 8. This prompted us to investigate the mechanism of apoptotic event.

**Results:**

Involvement of pro-apoptotic protein (Bax), active caspase-9 and cleavage of retinoblastoma protein was studied. Interestingly, the compounds caused upregulation of p21, check point proteins (Chk1, Chk2) and as well as their phosphorylated forms which are known to regulate the DNA damage pathway. Increased p53BP1 foci by immunolocalisation studies and TRF1 suggested the possible involvement of telomere and associated proteins in the apoptotic event. The telomeric protein such as TRF2 which is an important target for anticancer therapy against human breast cancer was extensively studied along with proteins involved in proper functioning of telomeres.

**Conclusions:**

The apoptotic proteins such as Bax, active caspase-9 and cleaved RB are up-regulated in the compound treated cells revealing the apoptotic nature of the compounds. Down regulation of TRF2 and upregulation of the TRF1 as well as telomerase assay indicated the decrease in telomeric length revealing telomeric dysfunction and thereby controlling the rapid rate of cell proliferation. In summary, chalcone-imidazolone conjugates displayed significant DNA damage activity particularly at telomeres and caused both apoptosis and senescence-like growth arrest which suggested that these compounds have potential activity against breast carcinoma.

## Background

MCF-7 is estrogen responsive breast cancer cell line and is widely used for studying compounds with anti-tumor activity for controlling the metastatic breast cancer [1]. Apoptosis or programmed cell death is a key regulator of growth control. With the advancement of cancer research the importance of apoptosis was realized in the context of response to anti-cancer compounds [2]. Unicellular organisms respond to DNA lesions by activating cell cycle check points and repair pathways, while multicellular organisms respond differently to DNA damage by eliminating the damaged cells through activating apoptosis. More over defects in DNA damage-induced apoptosis contribute to tumorigenesis [3] where as senescence is irreversible cell cycle arrest characterized by DNA double-strand breaks and activation of cell cycle check point responses leading to shortening of telomeres. More over, Senescence also involves expression of proteins such as p21 and p16 and the pathways that cause senescence will give a right path for effective cancer treatment [4,5].

The ends of chromosomes are protected by telomeres and have double stranded TTAGGG repeats of many kilobases with single stranded 3' over hang [6]. These double stranded telomeric DNA binding regions were bound with TRF1 and TRF2. Here TRF2 serves the protective function where as TRF1 regulates the telomeric length in a negative manner [7,8]. Recent reports have established TRF2 and its role in breast carcinoma [9], and highlights the importance of TRF2 in tumorigenesis. It was also observed that over expression of dominant negative form of TRF2 (DN-TRF2) in human cancerous cells caused a rapid loss of telomere leading to senescence or apoptosis [10-12]. Telomeric uncapping and replicative senescence triggers the DNA damage pathway in eukaryotes [13]. Cells react to double strand DNA breaks (DSBs), by initiating the DNA damage check response which temporarily pauses cell cycle progression until the damage has been repaired. The occurrence of DNA damage check point relies on the coordinated activites of the upstream kinases like ATM and ATR, the downstream transducer kinases CHK1 and CHK2, and the mediators such as p53BP1 and MDC1 [14-16]. The human cell cycle check point kinase 2 (Chk2) plays a vital role in the regulation of DNA damage response resulting in cell cycle arrest, DNA repair, apoptosis and senescence. Therefore screening and identification of drugs that can activate the check point proteins is considered as promising anti-cancer strategy, particularly in cases where apoptosis is blocked, cellular senescence can be induced as an alternative mechanism [17].

**Figure 1:**
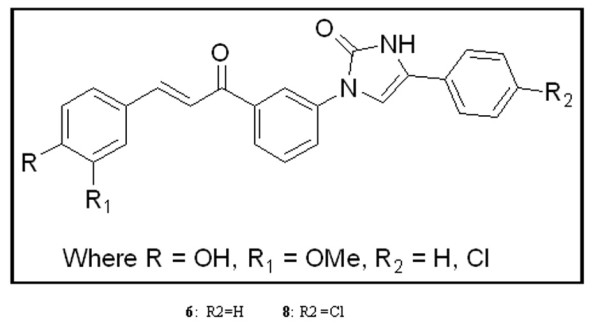
**Chemical structure of chalcone-imidazolone conjugates**. **6 **and **8 **represents chalcone-imidazolone conjugates 6 and 8.

Combretastatin (CA-4) compound contains structurally important feature, such as 2-phenyl rings separated by 2-carbon atom bridges and is a known inhibitor of tubulin polymerization [18-20]. Chalcone compounds are natural compounds isolated from edible plants and are structurally similar to CA-4 compound having 2-phenyl rings separated by 3-carbon atom bridges with inhibitory property against tubulin polymerization [21-23]. Imidazolones are also structurally similar to chalcones (2-phenyl rings are separated by 3-carbon atoms with 5 membered structures). Hence we focussed our attention towards preparing chalcone-imidazolone conjugates in search of potent anti-cancer molecules (Figure 1). Surprisingly, these conjugates have shown effective cytotoxicity in wide range of cancer cell lines [24].

In our previous studies [24] we have shown that these chalcone-imidazolone hybrids cause G2/M cell cycle arrest at 10 μM. But surprisingly the increased concentration of these conjugates to 30 μM caused accumulation cells in sub G1 phase (i.e G0 phase) indicated the apoptotic inducing nature of these conjugates. These interesting results prompted us to investigate their effect on the induction of DNA damage and controlling the rate of cell proliferation in MCF-7 cells involving proteins like check point proteins, telomere repeat binding factor 2 (TRF2), p53 binding protein1(53BP1) and other proteins associated with apoptosis. The present study also advocates the importance of the chalcone-imidazolone conjugates and its potential implication in anti-cancer therapy.

## Results

### Chalcone- imidazolone conjugates cause apoptosis in MCF-7 cells

**Figure 2:**
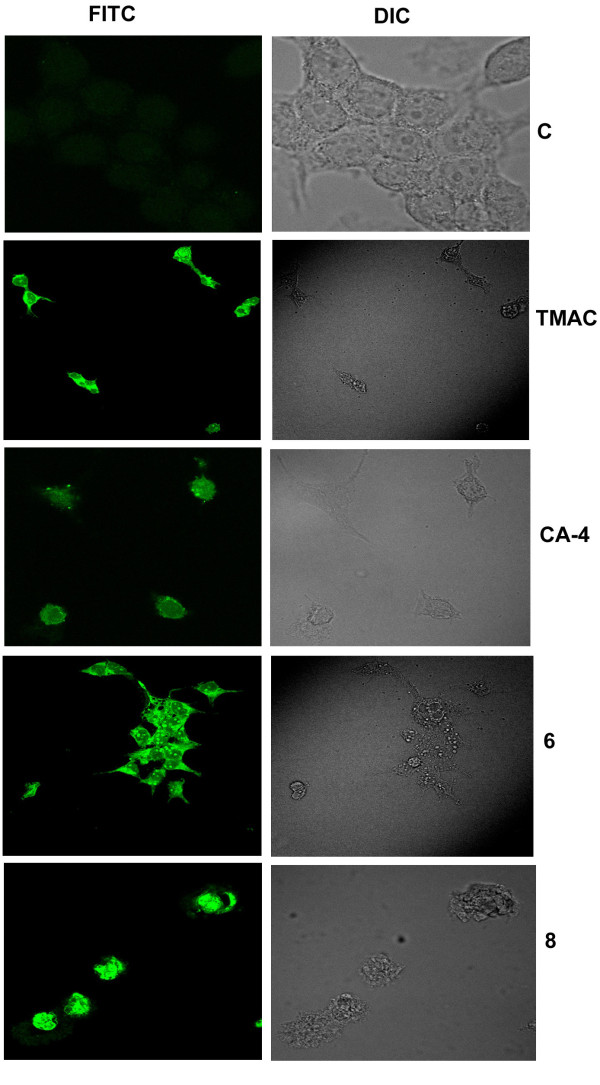
**Effect of chalcone-imidazolone conjugates in causing DNA fragmentation in MCF-7 cells**. MCF-7 cells were treated with compounds TMAC, CA-4, 6 and 8 at a concentration of 30 μM for a period of 24 h. The fragmented DNA was bind to antibody conjugated to FITC. The fluorescence was detected and imaged using confocal microscope. Untreated control cells, were not apoptotic and did not exhibit DNA fragmentation, where as the apoptotic cells in the compound treated condition show green coloured staining. The extent of apoptosis can be visualized by green staining and depicts the extent of DNA fragmentation. Here C denotes control untreated cells.

**Figure 3:**
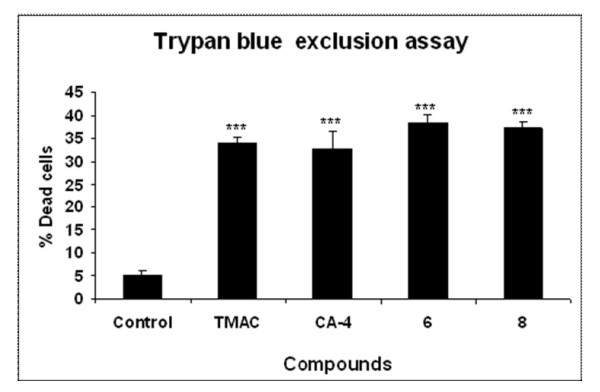
**Chalcone-imidazolone conjugates cause cell death in MCF-7 cells**. Trypan blue exclusion assay showing increase of cell death especially in conjugates 6 and 8. MCF-7 cells were treated at 30 μM concentration of TMAC, CA-4, 6 and 8 for 24 h. Each experiment was conducted for three times. The dead cells have lost the shiny nature and became dark blue coloured. Each experiment was conducted for three times. The p-values was found to be P < 0.001(***) when compared to control non-treated cells.

**Figure 4:**
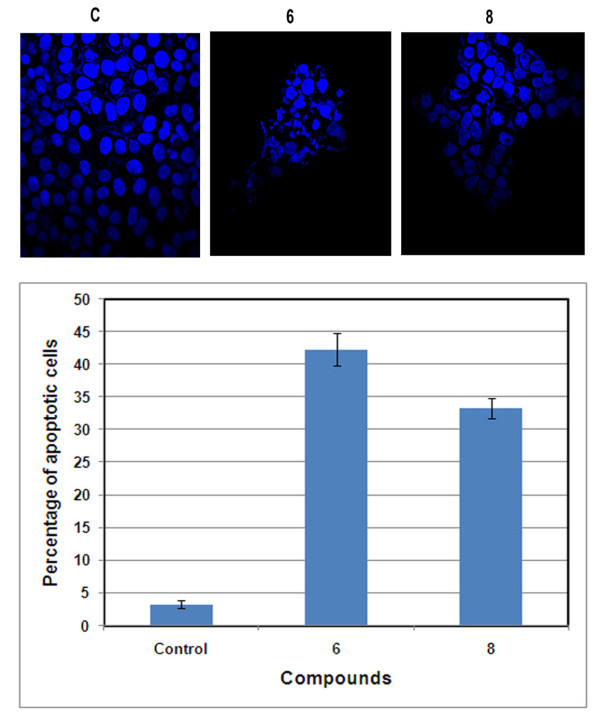
**Immunofluorescence studies to observe apoptotic related morphological changes in chalcone-imidazolone conjugates**. MCF-7 cells were grown in DMEM medium with 10% FBS for 12 h. Conjugates (**6 **and **8**) were treated at 30 μM for 24 h time period. The cells were then washed, fixed with 4% paraformaldehyde, and stained with nuclear dye DAPI. The slides were then examined by fluorescence microscopy and photographed. Cells with signs of apoptosis (fragmented nuclei) were observed in chalcone-imidazolone conjugates treated cells, when compared with control, non-treated cells.

In order to understand the apoptosis inducing ability of the compounds TUNEL assay was conducted. The data revealed that DNA fragmentation was more prominent in compound **6 **treated cells rather than in the case of the starting material TMAC (Figure 2). The apoptotic inducing nature of the compound was further validated by Trypan blue exclusion assay on cell viability. We observed that compound 6 and 8 treated cells to be less viable than control untreated cells, positive control (CA-4) and starting material (TMAC) (Figure 3). Blebbing, the characteristic feature of apoptosis was also clearly observed by nuclear staining with DNA dye DAPI in compound 6 and 8 after 24 h of compound treatment (Figure 4).

**Figure 5:**
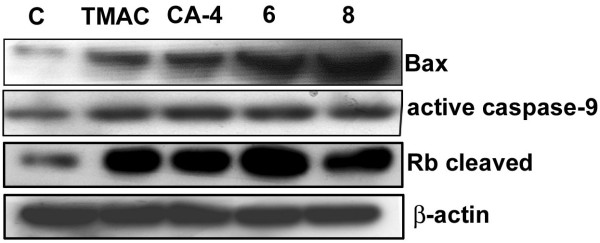
**Western blot analysis of proteins related to apoptosis**. MCF-7 cells at 80% confluence were treated with 30 μM chalcone-imidazolone conjugates for 24 h. 50 μg protein from total cell lysate was run on 10-15% SDS-PAGE and protein expression was detected by Western blotting. The expression of pro-apoptotic protein (Bax), active caspase-9 and the protein that undergo cleavage during apoptosis (Rb), the retinoblastoma protein was determined by using anti-Bax, anti-active caspase-9 and anti Rb-cleavage specific antibodies. β- actin was used as loading control. Here C represents control untreated cells.

During the process of apoptosis the up-regulation of proapoptotic protein (Bax) occurs as an early event which alone is sufficient to cause apoptosis [25]. MCF-7 cells do not contain endogenous caspase-3 and express caspase-9 during apoptosis. Retinoblastoma protein is a known and important substrate molecule that undergoes cleavage by the action of caspases into 48 and 68 kDa proteins [26] and ultimately leads to apoptosis. Hence we were interested to check whether chalcone imidazolone derivatives cause apoptosis by the involvement of Bax, active caspase-9 and cleavage of RB protein. Thus the MCF-7 cells were treated with compounds TMAC, CA-4, 6 and 8 at 30 μM concentration for 24 h and cell lysates were subjected to western blotting using specific antibodies. Our results have shown pronounced up regulation of Bax, active caspase-9 protein as well as cleaved RB in the compound treated cells in comparison to control untreated cells and thus confirming the apoptotic inducing nature of the compounds (Figure 5).

### Effect of Chalcone-imidazolone conjugates on DNA damage specific proteins (p53, Chk1and Chk2) in MCF-7 cells

**Figure 6:**
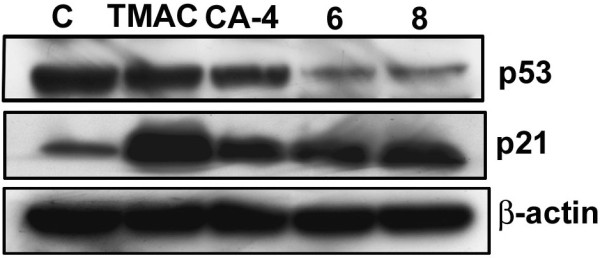
**Western blot analysis of tumour suppressor proteins (p53 and p21)**. MCF-7 cells were treated with chalcone-imidazolone conjugates for 24 h. The total protein extracts were prepared after 24 h and analysed with antibodies specific to tumour suppressor proteins such as p53 and p21. β-actin was used as loading control. The The molecular weights of p53, p21 proteins were 53 KDa, 21 KDa respectively.

DNA-damaging agents cause cell cycle arrest which ultimately results in apoptosis or senescence and will be regulated by either p53-dependent or independent pathways [27,28]. In order to determine the involvement of p53, p21 and p16 proteins in this event caused by chalcone-imidazolone conjugates, the cells were treated with compounds (TMAC, CA-4, 6 and 8) and Western blot analysis was carried out. The level of p53 protein was found to be down regulated while the levels of p21 and p16 was found to be up-regulated (Figure 6 and 12) particularly in compound 6 and 8 treated MCF-7 cells. The decreased levels of p53 and increased senescence like growth arrest as observed by up-regulation of p21 and p16 proteins clearly shows the lack of involvement of p53 in this event. Our results strongly support the existence of p53 independent pathway in senescence like growth arrest which has been earlier reported [29].

**Figure 7:**
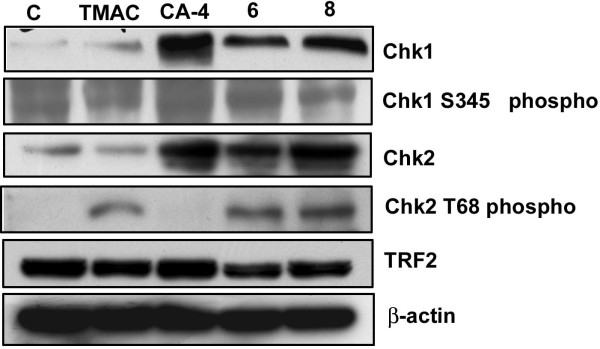
**Effect of chalcone imidazolone conjugates on DNA damage associated proteins**. MCF-7 cells treated at 80% confluency with conjugates TMAC, CA-4, 6 and 8 for 24 h. The total cell lysates were harvested and analyzed by immunoblotting with anti-Chk2, anti-Chk1, anti-phospho Chk2T^68^, anti-phospho Chk1S^345 ^and TRF2. The molecular weights of Chk2, Chk1, Chk2T^68^, Chk1S^345 ^and TRF2 were 61 KDa, 60 KDa, 61 KDa, 60 KDa and 65 KDa respectively. TMAC is the starting material and CA-4 is positive control. β-actin was used as a loading control. Western blots were representative of three independent experiments. The expression of Chk1, Chk2 and phosphorylated active forms of Chk2^T68 ^and Chk1S^345 ^were found to be up-regulated and levels of telomeric repeat binding factor 2 (TRF2) was found to be down regulated. C represents control untreated cells.

To concretely prove the role of check point proteins in the DNA damage, we have checked the levels of the Chk1 and Chk2 and as well as phosphorylated forms in the compound (6 and 8) treated cells. As expected we found the levels of the proteins were highly upregulated and involvement of these proteins in this damage event was highly elucidated (Figure 7).

### Effect of Chalcone-imidazolone conjugates on the telomeric binding protein TRF2 and p53BP1in MCF-7 cells

**Figure 8:**
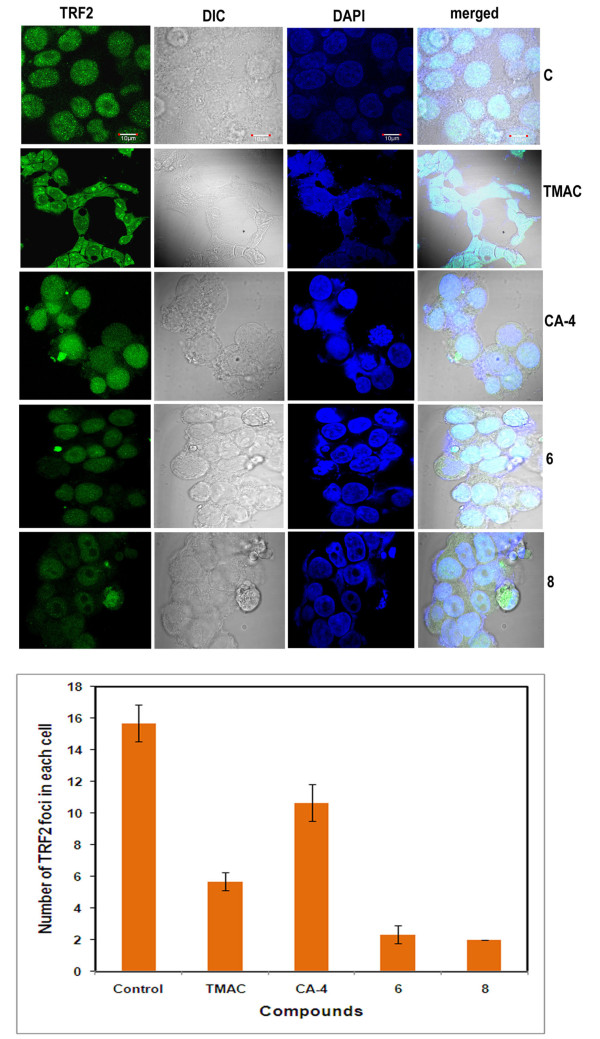
**Immunofluorescence based studies of telomere repeat binding factor 2 (TRF2) after treatment with chalcone- imidazolone conjugates**. Cells were grown in 10% FBS containing medium on cover slips and treated with 30 μM concentration of chalcone imidazolone compounds (6 and 8). TMAC, is the starting material and CA-4, is the positive control used. Cells were fixed and processed for immunofluorescence as described in materials and methods. Images were captured in confocal microscopy. The pattern of staining changes was also recorded. C represents control untreated cells.

**Figure 9:**
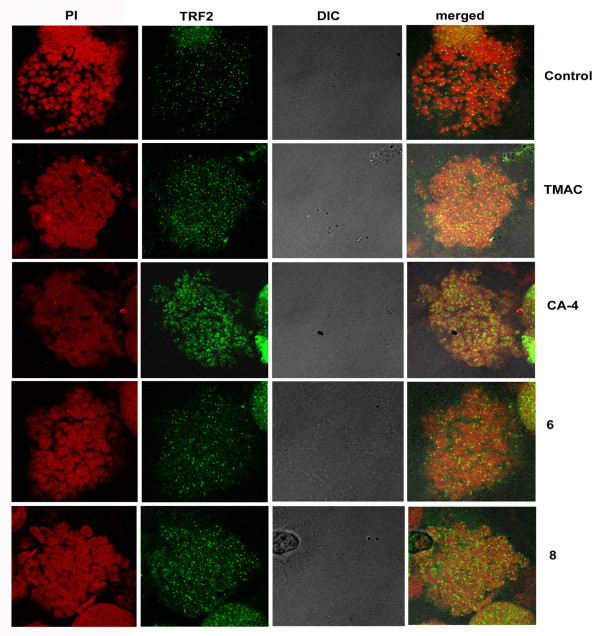
**Effect of Chalcone-imidazolone conjugates on size and distribution of TRF2 foci in metaphase spreads**. MCF-7 cells were synchronized with nocodazole (40 ng/ml) for 6 h time period followed by compound treatment (TMAC, CA-4, 6 and 8) at 30 μM final concentration for 24 h time period. This is followed by colchicine treatment (100 μg/m l) for 5 h and staining procedure was done with TRF2 antibody. Control represents untreated cells.

Telomeric-binding proteins like TRF2 play a fundamental role in maintaining the stability of telomere and controls DNA damage. Inhibition of TRF2 protein causes apoptosis or senescence at cellular level [10]. Thus we have speculated that chalcone-imidazolone compounds (6 and 8) might have a possible role on telomeric stability and regulate the expression of TRF2 protein. Cell lysates were prepared after treatment of cells with compounds for 24 h and subjected to western blotting using TRF2 specific antibody. As expected we found that the level of TRF2 was down regulated in compound 6 and 8 treated cells when compared to controls (Figure 7) and this was further confirmed by immunofluorescence studies. We observed that control untreated cells have shown a large number of TRF2 specific foci which were found to be drastically decreased in the cells treated with the compounds (Figure 8). Moreover in metaphase spreads of compound treated cells we have observed randomly distributed as well as fusion of TRF2 foci as shown in (Figure 9).

**Figure 10:**
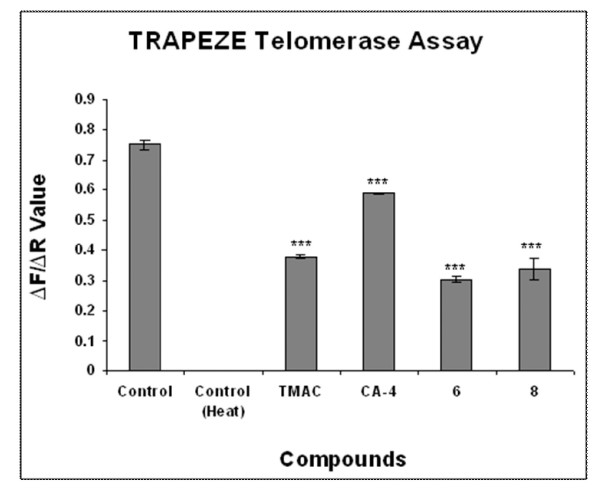
**Determination of telomerase activity in chalcone-imidazolone conjugates treated MCF-7 cells**. MCF-7 cells were treated with 30 μM concentration of chalcone imidazolone conjugates for 24 h and total cell lysates were subjected to telomerase specific PCR using fluorescently labeled primers of TRAPEZE XL telomerase detection kit. The telomerase activity was measured based on green fluorescence produced due to fluorescein (F). Internal control primers which are fluorescently labeled produces red colour which is represented by (R), due to sulforhodamine. Here TMAC is the starting material used for the synthesis of chalcone-imidazolone conjugates 6 and 8. CA-4, Combretastatin is positive control. Fluorescence reading due to fluorescein (F) was measured with excitation wave length of 495 nm and emission wave length of 516 nm. The fluorescence reading due to sulforhodamine (R) was measured at excitation wave length of 600 nm and emission wave length of 620 nm. The F/R ratio gives the telomerase activity. The p value for each compound was <0.001. The p value was generated by using student t-test by comparing the each compound vs control (untreated cells). Each experiment was conducted in triplicates.

Generally actively proliferating cancerous cells have more telomerase activity. Thus we have investigated the telomerase activity and conducted fluorescence based telomerase assay using cell extracts after compound (TMAC, CA-4, 6 and 8) treatment. Control untreated cells which serve as positive control has highest level of telomerase activity. Telomerase activity of the control cells is lost when the extract was heated at 85°C for 10 min. Fluorescence reading was taken as a measure of telomerase activity. To our surprise down regulation of telomerase activity was observed in compound 6 and 8 treated cells. Hence our data strongly supports the effect of compounds on telomere and regulate the DNA stability (Figure 10).

**Figure 11:**
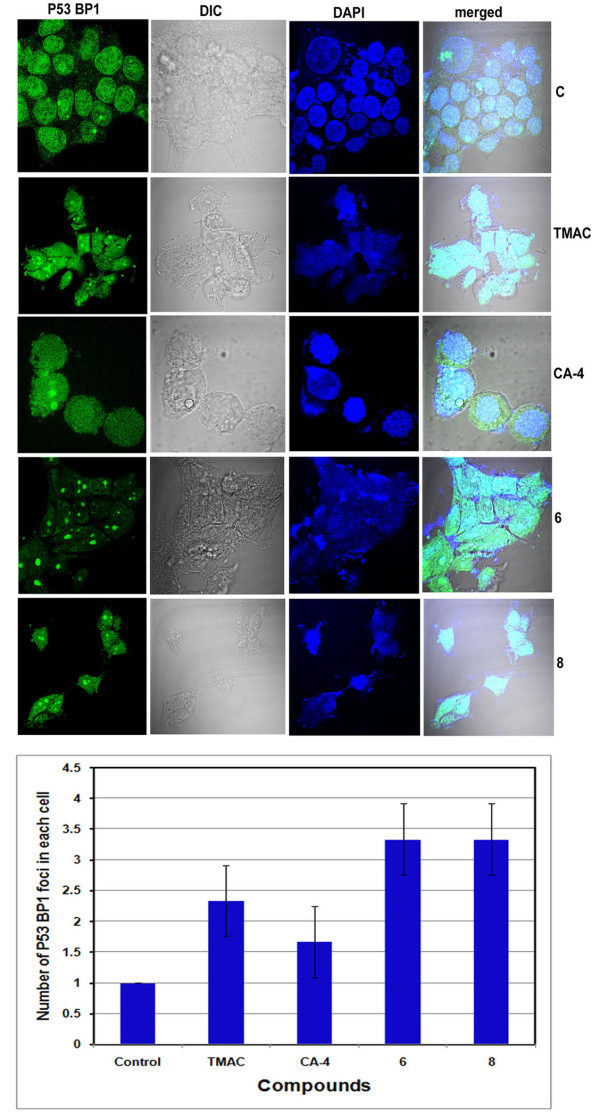
**Immunofluorescence based studies of p53BP1 after treatment with chalcone-imidazolone conjugates**. MCF-7 Cells were grown in 10% FBS containing medium on cover slips and treated with 30 μM concentration of chalcone imidazolone compounds (6 and 8). TMAC, is the starting material and CA-4, is the positive control used. Cells were fixed in 4% paraformaldehyde and processed for immunofluorescence with p53BP1 antibody as described in materials and methods. Images were captured using confocal microscopy. The pattern of staining changes was also recorded.

It was reported that uncapped telomeres are associated with DNA damage dependent early response factors known as p53BP1 and are the versatile index of telomeric dysfunction [30-32]. To ascertain the possible involvement of p53BP1 in the context of uncapped telomeres MCF-7 cells were treated with compounds (TMAC, CA-4, 6 and 8) and immunofluorescence experiments was carried out using p53BP1 specific antibody (Figure 11). The p53BP1 foci were found to be large and clearly seen in compound 6 and 8 treated cases, depicting the potential role of these compounds as anti-cancer molecules.

### Effect of Chalcone-imidazolone conjugates on telomeric genes in MCF-7 cells

**Figure 12:**
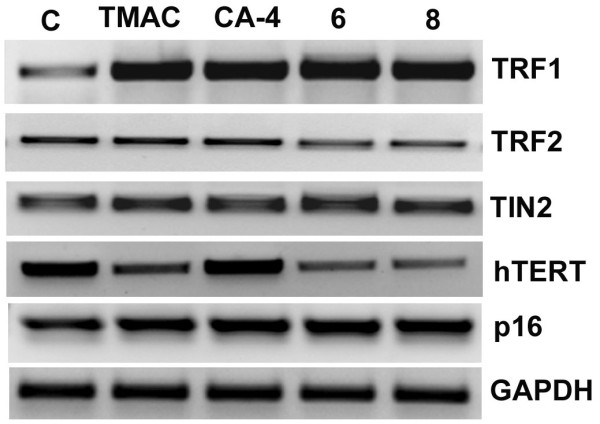
**mRNA levels of various genes involved in DNA damage at telomeres in MCF-7 cells**. MCF-7 cells were treated chalcone-imidazolone conjugates at a final concentration of 30 μM for 24h. After treatment total RNA was isolated and RT-PCR was conducted. The PCR products were separated on 1% agarose gel electrophoresis and visualized under U.V light. GAPDH was used as loading control. Each experiment was repeated three times. The gel pictures shown here were representative of three independent experiments.

The results obtained on TRF2 protein in compound treated cells prompted us to study the role of important complex of proteins (TRF1, TRF2, TIN2 and hTERT) that are closely associated with telomere stability and functioning. Here the MCF-7 cells were treated with chalcone-imadazolone conjugates, RNA was isolated and RT-PCR analysis has been carried out to observe the mRNA levels of TRF1, TRF2, TIN2 and hTERT. The levels of TRF1 and TIN2 were found to be upregulated in all the compounds tested where as the levels of TRF2 and hTERT was decreased in case of compounds 6 and 8. Thus our data clearly reveals the effect of chalcone imidazolones on telomeric complex and cause DNA damage (Figure 12).

## Discussion

We have prepared hybrid molecules involving both chalcones, the inhibitors of tubulin polymerization and imidazolones, the cytotoxic compounds with the aim of improving the effective cytotoxicity against various cancers that cause mortality of human beings. Breast cancer is one of the most prevalent cancers in the world and many are diagnosed with this disease every year [1]. NCI data against these chalocone-imidazolone compounds were tested and found that these compounds are effective against certain cell lines such as MCF-7 breast carcinoma. Henceforth it is very important to study these molecules in this cell line. Previous studies in this laboratory [24] have shown that chalcone-imidazolone conjugates cause significant apoptosis at 30 μM concentration. But in depth molecular mechanism involved has not been studied.

Cells arrested and which cannot repair DNA damage would undergo death by processes called as apoptosis or senescence, a growth arrest phenomena. Apoptosis is a type of programmed cell death (PCD), the abrogation of this process leads to carcinogenesis [34,35] and is characterized by membrane blebbing, cell shrinkage, nuclear condensation and DNA fragmentation [36,37].

Tunel assay clearly revealed the apoptosis inducing ability of the compounds (6, 8) when compared to controls (Figure 2). More over the cell death due to conjugates 6 and 8 was further confirmed by experiments such as Trypan blue exclusion assay, (Figure 3) which gives percentage of cell death in a population of cells as well as membrane blebbing and nuclear condensation which are the characteristics of apoptosis by DAPI nuclear staining (Figure 4). Bax and active caspase-9 are crucial regulator of apoptosis where as cleavage of Rb is the indicator of apoptosis [25,26]. Our results have shown pronounced up regulation of Bax protein, cleaved caspase-9 and cleaved Retinoblastoma (Rb) in the compound treated cells in comparison to control confirming the apoptotic inducing nature of the compounds (Figure 5).

P53 is a classical tumor suppressor protein and universal sensor of genotoxic stress that functions by inhibiting cell proliferation [38]. In general DNA-damaging agents cause cell cycle arrest which finally results in apoptosis or senescence and will be regulated by either p53-dependent or independent pathways [27,28]. Our results strongly support the existence of p53 independent pathway (Figure 6) and senescence like growth arrest which has been reported earlier [29]. Studies have shown that Chk2 dependent senescence as well as transcriptional activation of p21 will contribute to the tumour suppression in the p53-defective SK-BR3 breast carcinoma [32]. Thus we have focused our attention on check point 2 (Chk2) as an alternative mechanism. Dysfunctional telomeres resulted in phosphorylation and activation of Chk2 in ATM dependent manner which ultimately leads to permanent arrest in cell cycle which is termed as replicative senescence. There are also reports that only senescent cells possessed Chk1 and Chk2 phosphorylated at amino acid residues S345 and T68 respectively and studies by [33] also suggested that some of the drugs also can activate Chk1. To concretely prove the role of check point proteins in the DNA damage induced senescence, we have checked the levels of the Chk1 and Chk2 and as well as phosphorylated forms in the compound treated cells. As expected we found the levels of the protein were highly upregulated and involvement of these proteins in this event was highly elucidated (Figure 7)

The role of Chk2 in this DNA damage event prompted us to check the Telomeric-binding protein 2 (TRF 2), whose inhibition causes apoptosis or senescence at cellular level by causing DNA damage response [10,32]. Thus we hypothesized that chalcone imidazolone compounds (6 and 8) might have a possible role on telomeric stability by regulating the expression of TRF2 protein. Hence cell lysates were subjected to western blotting using TRF2 specific antibody and we found that the levels of TRF2 were decreased in compound treated cells (Figure 7). This was further supported by immunolocalisation studies using TRF2 antibody, where in we observed, decreased number and as well fusion of TRF2 foci (Figure 8, 9). Thus our results have shown a way to potentially inhibit proliferation of breast cancer cells and TRF2 might be effective drug target.

Further we continued our studies on telomerase activity and its role in breast cancer progression [9]. We have experimented towards measuring telomerase activity in the context of compound treatment. To our surprise down regulation of telomerase activity was observed in compound 6 and 8 treated cells. Hence the data strongly supports TRF2 dependent DNA stability (Figure 10). Previous studies have proved that the uncapped telomeres are found to be associated with p53BP1 [30-32]. To ascertain the possible involvement of p53BP1 in the context of uncapped telomeres MCF-7 cells were treated with compounds (TMAC, CA-4, 6 and 8) and were subjected to immunofluorescence experiments using p53BP1 specific antibody (Figure 11). We found the formation of large foci in compound TMAC, 6 and 8 treated cells, indicating the potential role of this compound as anti-cancer molecule.

Both in vivo and in vitro studies [12] have shown that TRF2 inhibition limits cell proliferation of the human cancer cells and can be considered as an important target for the development of anti-cancer therapeutics. TRF1 and TRF2 are the two important molecules which regulate the telomeric length and DNA damage respectively. TRF1 negatively regulates the telomere length and TRF2 is actively involved in the protection of telomeres. Recent reports have shown that telomere shortening triggers senescence which requires the active role of p21 but does not need p16 activity [39]. The observations made from the immunolocalisation studies of the TRF2 have shown the importance of this protein in telomeric stability during DNA damage. So we studied the catalytic subunit of telomere (hTERT) and the important telomeric binding proteins such as TRF1, TRF2, TIN2 and their expression patterns. RT-PCR results have indicated increased mRNA levels of TRF1 and TIN2. While the levels of TRF2 and hTERT was decreased in case of compound 6 and 8 treated cells, Thus our data clearly reveals the effect of chalcone imidazolones on telomeric complex and cause DNA damage (Figure 12).

## Conclusions

Studies on effect of chalcone imidazolone compounds on MCF-7 cells identified the apoptotic as well as senescence inducing nature of the compound. As a part of cell cycle arrest the levels of p53, p21 and p16 was studied. Telomeres binding protein factor 2 (TRF2) as well p53BP1 that are associated due to DNA damage initiated telomeric uncapping was highly elucidated. Certain key molecules that regulate the DNA damage pathway such as Chk2, Chk2T^68^, Chk1and Chk1S^345 ^was found to be activated and regulate the senescence as well as ultimate apoptotic pathway which occurred by activated levels of Bax, active caspase-9 and cleaved Rb. This data strongly supports the caspase dependent apoptotic pathway which was published previously [24]. Ultimately our data has extensively and clearly shown telomere initiated DNA damage which involves both senescence like growth arrest and ultimately apoptosis.

## Methods

### Cell culture

The human breast cancer cell line MCF-7 was purchased from American Type culture collection was maintained in Dulbecco's modified Eagle's medium (DMEM) (Invitrogen), supplemented with 10% fetal calf serum and 100U/ml Pencillin and 100 mg/ml streptomycin sulfate (Sigma). The cell line was maintained at 37°C in a humidified atmosphere containing 5% CO_2 _in the incubator.

### Tunel assay

Tunel assay was conducted by using the Apoalert DNA fragmentation Assay kit (Clone tech) according to manufacturer instructions. This kit detects the apoptosis-induced nuclear DNA fragmentation via fluorescence based assay. The assay is based on the principle of terminal deoxy nucleotidyl transferase (TdT)-mediated dUTP nick-end-labelling (TUNEL) method. TdT catalyzes incorporation of fluorescein-dUTP at the free 3'-hydroxyl ends of fragmented DNA. Flourescein-labeled DNA can be detected via confocal microscopy.

### Immunofluorescence

MCF-7 breast cancer cells were seeded on cover slips and treated with chalcone imidazolone compounds at concentration of 30 μM for 24 h. After treatment, cover slips were fixed with a paraformaldehyde solution (4% in 1X PBS) for 20 min at room temperature. Cell permeabilization was achieved by administration of Triton X-100 solution (0.2% in 1X PBS) for 5 min. Then cover slips were kept in 100% methanol at 4°C over night. Subsequently, cover slips were blocked with a 1% BSA solution for 60 min and then incubated with anti TRF2 and P53BP1 (1:100) antibody at room temperature for 2 h. The slides were washed three times each of 5 min with PBST. Then cover slips were incubated with a FITC-conjugated anti-rabbit secondary antibody (Jackson Immuno Research Laboratories Inc., Pennsylvania, USA) for one hour and cover slips were washed three times with PBST solution and mounted with DAPI/PI solution. Finally, cells were observed under confocal microscope (Olympus FV1000). Images taken were processed with the support of the flow view version 1.7c software program.

### TRAPeze XL Telomerase assay

This detection kit (Millipore) is a sensitive as well as rapid PCR based fluorescent assay for detecting telomerase activity in cell extracts. Treatments were given for 24 h with compounds TMAC, CA-4, 6 and 8 at a concentration of 30 μM. The cell lysis was carried out using CHAPS buffer. The extracts were further used for PCR reaction. Heat inactivated control untreated MCF-7 cell extract, which lost the telomerase activity is a negative control as well as control non-heat inactivated cell extract which has efficient telomerase activity was used as positive control. Here the telomerase master mix which contains fluoro labeled primers designed both for Telomerase as well as internal control TSK2 template was used along with cell extract and Taq polymerase. The telomerase activity was measured with green fluorescence (F). Internal control amplification pattern was indicated by sulphorhodamine (R), which gives red colour. The ratio of F and R gives the actual telomerase activity. The conditions of PCR reaction conditions were followed according to the manufacturer's recommendation.

### Protein extraction and Western blot analysis

Total cell lysates from cultured MCF-7 cells were obtained by lysing the cells in ice-cold RIPA buffer (1XPBS, 1% NP-40, 0.5% sodium deoxycholate and 0.1% SDS) and containing 100 mg/mL PMSF, 5 mg/mL Aprotinin, 5 mg/mL leupeptin, 5 mg/mL pepstatin and 100 mg/mL NaF. After centrifugation at 12,000 rpm for 10 min, the protein in supernatant was quantified by Bradford method (BIO-RAD) using Multimode varioskan instrument (Thermo-Fischer Scientifics). Fifty micrograms of protein per lane was applied in 12% SDS-polyacrylamide gel. After electrophoresis, the protein was transferred to polyvinylidine difluoride (PVDF) membrane (GE Biosciences). The membrane was blocked at room temperature for 2 h in TBS + 0.1% Tween20 (TBST) containing 5% blocking powder (Santacruz). The membrane was washed with TBST for 5 min, primary antibody was added and incubated at 4°C overnight (O/N). p53, p21, Chk2, Chk2 T^68^, Chk1, Chk1 S^345^, Bax, active caspase-9 Cleaved Rb antibodies were purchased from Imgenex, USA. TRF2 and p53BP1 antibodies were purchased from Cell Signalling Company. The membrane was incubated with corresponding horseradish peroxidase-labeled secondary antibody (1:2000) (Santa Cruz) at room temperature for 1 h. Membranes were washed with TBST three times for 15 min and the blots were visualized with chemiluminescence reagent (Thermo Fischer Scientifics Ltd.). The X-ray films were developed with developer and fixed with fixer solution (Kodak Company Ltd).

### Nuclear staining

MCF-7 cells were seeded on cover slips, treated with compounds for 24 h, washed with PBS and fixed with 4% Paraformaldehyde for 15 min at room temperature. Fixed cells were incubated in PBS (pH 7.4) containing DNAse-free RNase (Sigma) for 30 min at 37°C and stained with DAPI. Nuclear morphology of the cells was observed under confocal microscope.

### Trypan blue exclusion test of cell viability

This method is used to determine the number of viable cells present in cell suspension. This method is based on the principle that live cells possess intact cell membranes that excludes trypan blue; whereas dead cells are not capable of excluding trypan blue. Here viable cells show a clear cytoplasm where as non viable cells show blue colour cytoplasm. Compounds were treated for 24 h time period. After compound treatment (TMAC, CA-4, 6 and 8) at 30 μM concentration the cells were trypsinised and the dead and viable cells were counted. Each sample was assayed for triplicates. In this assay 10 μl of 0.4% solution of trypan blue was added to 100 μl of cells. Once after mixing the sample was loaded on to haemocytometer and examined immediately. The percentage of dead cells was calculated.

### Semi-quantitative reverse transcription PCR (RT-PCR)

Total RNA was extracted using RNeasy mini kit (Qiagen, USA) and reverse transcribed into cDNA using superscript II reverse transcriptase (Invitrogen life technologies). The PCR was carried out with specific primers (Table 1) in Takara Bioscience PCR machine. The products were electrophoresed on agarose gel (1%) followed by staining with ethidium bromide and visualized under U.V. light. The signal intensity of respective bands was measured by means of the quantity one version 4.1.1 soft ware using BIORAD image analysis system (CA, USA).

**Table 1:** Primer sequences used in Reverse-transcription polymerase chain reaction (RT-PCR)

S.NO	Primer	Primer Sequence	Primer length	**Tm(**^**0**^**C)**
**1**.	**hTERT, Size: 214bp**			
	**Sense primer**	**5'-cgtggtttctgtgtggtgtc-3'**	**20**	**60**
	**Anti sense primer**	**5'-ccttgtcgcctgaggagtag-3'**	**20**	**60**
**2**.	**TRF2, Size: 160bp**			
	**Sense primer**	**5'-gtacccaaaggcaagtggaa-3'**	**20**	**59.97**
	**Anti sense primer**	**5'-tgacccactcgctttcttct-3'**	**20**	**59.99**
**3**.	**TRF1, Size:212bp**			
	**Sense primer**	**5'-tctctctttgccgagctttc-3'**	**20**	**59.84**
	**Anti sense primer**	**5'-ggctgattccaagggtgtaa-3'**	**20**	**59.93**
**4**.	**TIN2, size: 208bp**			
	**Sense primer**	**5'-ctgagcccatggaacagaat-3'**	**20**	**60.67**
	**Anti sense primer**	**5'-tccttatggcctcccctagt-3'**	**20**	**59.92**
**5**.	**P16, size: 171bp**			
	**Sense primer**	**5'-gggtcgggtagaggaggtg-3'**	**19**	**61.85**
	**Anti sense primer**	**5'-gcgctacctgattccaattc-3'**	**20**	**59.67**

### Immunocytochemistry for metaphase spreads

MCF-7 cells were grown in 60 mm dish till 70% confluency. Nocodazole (40 ng/ml) was added 12 h before the compound treatments (TMAC, CA-4, 6 and 8) at 30 μM final concentration. Treatment with compounds was carried for 24 h time period. 4-5 h prior to harvest 100 μg/ml colchicine was added. Immediately after treatment cells were washed with PBS and trypsinised. Then cell pellet was dissolved 0.56% KCl and incubated for 20 min followed by centrifugation at 1000 rpm for 3 min. Supernatant was discarded. The pellet is flicked and nuclei solution was added in to funnel and cytospin was carried out at 1000 rpm for 5-6 min. Slides were removed and fixed in 4% paraformaldehyde. Slides were washed with PBS for 15 min and incubated in triton X-100 and sodium azide solution for 45 min. This is followed by addition of 30 μl of TRF2 antibody and incubated overnight at 4°C. The slide is incubated in blocking solution (goat serum) for 30 min at 4°C. Secondary antibody was added and incubated for 3 h in dark at room temperature. Slides were washed for 15 min and incubated with RNaseA (0.5 μg/ml) and mounted in PI (Vecta shield).

### Statistical Analysis

Statistical Analysis was performed using the graph pad software to evaluate the significant difference between the control and treated samples. All variables were tested in three independent experiments. The results were reported as mean ± SD. * represents *p*-value < 0.05, ** represents *p*-value < 0.01, *** represents *p*-value < 0.001.

## Competing interests

The authors declare that they have no competing interests.

## Authors' contributions

MJR performed the immunofluorescence staining, RT-PCR and designed the protocol. SNCVLPV did the western blotting. PS and DM carried out the cell culture experiments. GRK synthesized the compounds. UB and MPB designed the experiments, analyzed the data and prepared the manuscript. AK designed the experiments for synthesis of the hybrid molecules and revised the manuscript.

All authors read and approved the final manuscript.
